# Diagnostic and therapeutic dilemma in Stevens–Johnson syndrome-like acute graft-versus-host disease after liver transplantation: A case report

**DOI:** 10.3389/fimmu.2022.917782

**Published:** 2022-08-18

**Authors:** Yi-Teng Hung, Yau-Ren Chang, Hsuan-Ning Wang, Wei-Chen Lee, Chen-Fang Lee, Chun-Bing Chen

**Affiliations:** ^1^ Drug Hypersensitivity Clinical and Research Center, Chang Gung Memorial Hospital, Linkou, Taipei and Keelung, Taiwan; ^2^ Department of Dermatology, Chang Gung Memorial Hospital, Linkou, Taiwan; ^3^ Department of Surgery, Chang Gung Memorial Hospital, Linkou, Taiwan; ^4^ Department of Dermatology, New Taipei Municipal TuCheng Hospital, New Taipei City, Taiwan; ^5^ Department of Liver and Transplantation Surgery, Chang Gung Memorial Hospital, Linkou, Taiwan; ^6^ School of Medicine, College of Medicine, Chang Gung University, Taoyuan, Taiwan; ^7^ Cancer Vaccine and Immune Cell Therapy Core Laboratory, Chang Gung Memorial Hospital, Linkou, Taiwan; ^8^ Chang Gung Immunology Consortium, Chang Gung Memorial Hospital and Chang Gung University, Taoyuan City, Taiwan; ^9^ Department of Dermatology, Xiamen Chang Gung Hospital, Xiamen, China; ^10^ Whole-Genome Research Core Laboratory of Human Diseases, Chang Gung Memorial Hospital, Keelung, Taiwan; ^11^ Immune-Oncology Center of Excellence, Chang Gung Memorial Hospital, Linkou, Taiwan; ^12^ Graduate Institute of Clinical Medical Sciences, College of Medicine, Chang Gung University, Taoyuan, Taiwan; ^13^ Genomic Medicine Core Laboratory, Chang Gung Memorial Hospital, Linkou, Taiwan; ^14^ School of Medicine, National Tsing Hua University, Hsinchu, Taiwan

**Keywords:** anti-TNF-α, graft-versus-host disease, immunomodulant, Stevens-Johnson syndrome, target therapy, liver transplantation

## Abstract

**Background:**

Acute graft-versus-host disease (aGVHD) is a severe and fatal complication after orthotopic liver transplantation (OLT). Clinical manifestations of severe aGVHD can resemble drug-induced Stevens–Johnson syndrome (SJS)/toxic epidermal necrolysis (TEN), and there are also various medications, such as antibiotics and immunosuppressants, used after transplantation, causing a diagnostic dilemma. Furthermore, there have been no standardized diagnostic and therapeutic strategies for OLT-aGVHD due to its rarity.

**Case summary:**

A 52-year-old man presented with generalized maculopapular eruptions, fever, and pancytopenia 1 month after OLT and 4 days after taking sulfamethoxazole/trimethoprim. After assessment of the scoring criteria for drug causality of drug allergy, histopathological findings of skin biopsy, lymphocyte activation test of the potential offending drug, and microchimerism study, the diagnosis was in favor of aGVHD mimicking SJS/TEN. Considering severe sepsis, the anti-tumor necrosis factor alpha (TNF-α) agent, etanercept, was used to replace tacrolimus and corticosteroid. Skin lesions resolved gradually after anti-TNF-α biologics rescue; tacrolimus and corticosteroid therapy were re-administrated after controlling sepsis. Pancytopenia recovered and the patient was discharged in a stable condition.

**Conclusion:**

We demonstrated a diagnostic strategy for OLT-aGVHD. Targeting therapy with anti-TNF-α blockade and a temporary withdrawal of traditional immunosuppressants may be among effective and safe therapeutic options of OLT-aGVHD for those with severe sepsis.

## Introduction

Graft-versus-host disease (GVHD) is defined as a reaction of donor immune cells against recipient tissues. Any medical procedure transferring viable allogeneic lymphocytes into a recipient has a risk of the occurrence of GVHD. GVHD in solid organ transplantation is less common than in hematopoietic stem cell transplant (HSCT). Among solid organ transplants, GVHD takes place more likely in recipients of liver and intestinal transplantation due to plenty of donor lymphocytes (1). In 1988, Burdick et al. firstly reported this fatal complication after orthotopic liver transplantation (OLT), and increasing cases have been described since then (2, 3). The incidence of GVHD after OLT has been estimated at 0.1–2% (4).

Acute GVHD (aGVHD), characterized by abnormalities of the skin, gastrointestinal (GI) tract, and liver, can be divided into classic form (occurring within the first 100 days after transplantation) and late-onset form (occurring with similar acute features but after 100 days of transplantation). The clinical presentations of OLT-associated aGVHD are similar to those of HSCT-associated aGVHD, including fever, skin rash, GI symptoms (i.e., bloody diarrhea), pancytopenia, and accompanied sepsis. In contrast, impaired liver function in OLT-associated aGVHD is not as common as HSCT-associated aGVHD (3, 5). Adverse drug reactions and opportunistic viral infections, such as cytomegalovirus (CMV) or Epstein–Barr virus (EBV) infection, are often encountered after transplantation, and their cutaneous manifestations are sometimes difficult to be differentiated from aGVHD (3, 6). Of note, severe aGVHD can share a clinical resemblance to drug-induced Stevens–Johnson syndrome (SJS)/toxic epidermal necrolysis (TEN), causing a diagnostic dilemma (7). It is crucial to differentiate between these two diseases because the managements are largely different. Due to insufficient large-scale studies and a rarity, the treatments of OLT-associated aGVHD have not been standardized. However, the mortality of aGVHD after OLT remains extremely high, especially in coexistence with infection, and ranges from 85% to 90% (8).

Herein, we reported a case of aGVHD after OLT presenting as SJS/TEN-like eruptions complicated with severe infection and neutropenia. A comprehensive survey and appropriate management with targeting therapy were demonstrated to solve the dilemma.

## Case description

**Figure 1:**
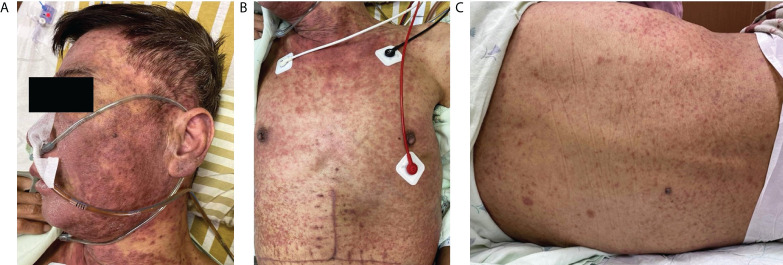
Skin manifestations. **(A)** Diffuse erythematous–violaceous maculopapular exanthems on the face. **(B)** Stevens–Johnson syndrome-like atypical flat targets on the neck. **(C)** Erythematous folliculocentric papules on the back.

A 52-year-old man with alcoholic liver cirrhosis, Child-Pugh score C, received deceased donor liver transplantation at our institution in January 2021. The post-transplant course was uneventful initially. However, 4 weeks after transplantation, dysuria happened during the regular follow-up in the clinic and Baktar^®^ (sulfamethoxazole 400 mg/trimethoprim 80 mg) was prescribed. Skin rashes were noted on his face and upper trunk with fever and refractory diarrhea developed 4 days later. There was no clinical history of drug reactions before transplantation. He was admitted to ward for the tentative diagnosis of drug-induced allergic reaction or aGVHD. Physical examination showed generalized erythematous-to-violaceous maculopapular eruptions on the face, ears, and upper trunk (**Figure 1A**), sparing upper extremities along with palms and soles. The percentage of total body surface area of erythematous rashes was 25%. Moreover, atypical flat target lesions that were characteristic for SJS/TEN were noted on the neck and trunk (**Figure 1B**); erythematous folliculocentric papules developed on the back (**Figure 1C**). Oral mucositis and conjunctivitis were also noticed. The laboratory examinations revealed severe neutropenia (white blood cell: <0.1 × 10^9^ cells/L, neutrophil: 40%), anemia (hemoglobin: 7.8 g/dl), thrombocytopenia (platelet count: 25,000 × 10^9^ cells/L), and renal dysfunction (creatinine: 2.35 mg/dl) with normal liver function. Immunosuppressants with oral prednisolone (15 mg/day) and tacrolimus (Prograf ^®^) (12 mg/day) to prevent aGVHD were discontinued due to severe sepsis and neutropenic status. The trough level of tacrolimus, FK506, was within an acceptable range. Granulocyte-colony stimulating factor (300 mg/day) was prescribed for 3 days to treat neutropenia. He was transferred to intensive care unit subsequently due to septic shock with bacteremia. Blood cultures grew *Escherichia coli* (*E. coli*). Mechanical ventilator support was used afterwards for respiratory failure and consciousness disturbance.

**Figure 2:**
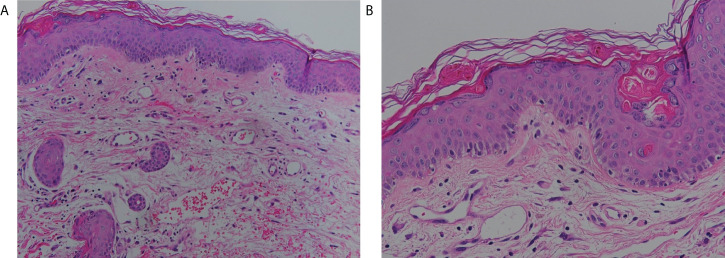
Histological features. Skin biopsy demonstrated mild exocytosis, basal layer vacuolization, lymphocyte infiltrate at the interface, and mild melanin incontinence (hematoxylin and eosin stain, **A**: 200×, **B**: 400×).

Due to the possibility of adverse drug reaction, the scoring systems for assessment of drug causality were used to evaluate the association of sulfamethoxazole/trimethoprim and SJS/TEN. There were no other suspicious offending drugs noted after reviewing his drug history in the recent 2 months. The algorithm of drug causality for epidermal necrolysis (ALDEN) score was three points (classified as “possible”) and Naranjo algorithm score was three points (classified as “possible”) for sulfamethoxazole/trimethoprim. Since drug-induced SJS/TEN could not be ruled out, an effective targeting therapy with anti-tumor necrosis factor alpha (TNF-α) blockade with etanercept (Enbrel^®^), which was effective for both SJS/TEN and aGVHD (9–12), was initiated at two doses of 50 mg twice a week. Skin biopsy was performed on the neck, and the histopathology showed exocytosis, dyskeratotic cells, basal layer vacuolization, interface lymphocytic infiltrates, mild melanin incontinence, and papillary dermal edema (**Figure 2**). The histopathological features showed mild lichenoid dermatitis and was consistent with GVHD. The granulysin- and granzyme B-based lymphocyte activation tests (13) both showed negative response to sulfamethoxazole/trimethoprim. Human leukocyte antigen (HLA) genotyping revealed an absence of HLA-B*13:01, HLA-B*15:02, HLA-B*38:02 and HLA-B*39:01, which were identified as a significant genomic indicator for drug-induced severe cutaneous adverse reactions, including SJS/TEN (14). Serologic diagnosis of *Mycoplasma pneumonia*, EBV, and herpes simplex virus (HSV) showed no evidence of recent infection, and CMV detection by polymerase chain reaction of plasma revealed negative results. Furthermore, the chimerism study [short tandem repeat (STR) from blood sample] showed that 19% of HLA was originated from the donor, supporting the diagnosis of aGVHD. The lymphocyte subpopulations by flow cytometry showed mild increased CD3 (T cell) (88.4% to 93.9%) (normal, 50%–85%), constant CD19 (B cell) (3.5% to 3.3%) (normal, 7.8%–22.8%), decreased CD4 (+) T cell (70.2% to 42.4%) (normal, 23%–53%), increased CD8 (+) T cell (21.9% to 44.5%) (normal, 19%–49%), and decreased CD4/CD8 (3.2 to 0.9) (normal, 0.59–2.24) compared to 1 week before the occurrence of aGVHD.

**Figure 3:**
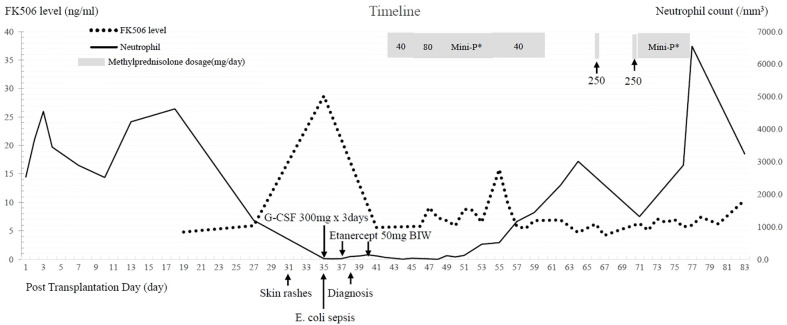
Clinical course after liver transplantation. FK506: tacrolimus, *E. coli*: *Escherichia coli*, G-CSF: granulocyte colony-stimulating factor Mini-P*: Methylprednisolone mini-pulse therapy with dose tapering-methylprednisolone 200 mg divided in four doses per day, then 160 mg, 120 mg, and 80 mg, followed by 40 mg divided by two doses per day.

Though leukopenia remained, the skin lesions resolved gradually in 1 week after etanercept treatment. One week later, the infectious problem was under control following treatments of broad-spectrum antibiotics (meropenem and teicoplanin), adequate critical care, and strict contact isolation. We subsequently prescribed methylprednisolone (solu-Medrol^®^) at a low dose of 40 mg/day to 80 mg/day for 1 week, then initiated a gradual tapering of mini-pulse corticosteroid therapy (methylprednisolone 200 mg, and then 160 mg, 120 mg, 80 mg, 40 mg per day). We also added back tacrolimus at an initial dose of 2 mg/day to suppress the overexpression of donor immunity. Pancytopenia was gradually reversed and the repetitive microchimerism examination revealed less than 1% presentation of donor HLA. Mini-pulse methylprednisolone therapy with a single dose of 250 mg was intermittently used when residual symptoms of aGVHD relapsed. After 4 months, he was discharged in a stable condition, and only low-dose immunosuppressants (oral prednisolone at a dose of 5 mg/day and tacrolimus at a dose of 6 mg/day) were needed for the patient. The clinical course is summarized in **Figure 3**.

## Discussion

In this case, we demonstrated the diagnostic and therapeutic strategies for a case of OLT-aGVHD mimicking adverse drug reaction as SJS/TEN, complicated with severe infection and neutropenia. A series of workup to exclude the possibility of drug adverse reactions (especially SJS/TEN) and viral infections are mandatory to diagnose OLT-aGVHD. For the individuals with aGVHD suffering from severe sepsis and pancytopenia, administration of corticosteroid-sparing targeting agents, such as anti-TNF-α biologics with etanercept, and a temporary withdrawal of traditional immunosuppressants may serve as an optimal therapeutic option. Further traditional immunosuppressants for GVHD, including corticosteroids, can be re-administrated after well control of infection and pancytopenia.

Drug-induced SJS/TEN and aGVHD presenting as SJS/TEN have been both reported in liver transplant recipients (7, 15). Multiple medicines are often used in the post-transplant patients, including nonsteroidal anti-inflammatory drugs (NSAIDs), antibiotics, and immunomodulants. Of note, NSAIDs and antibiotics are also common potential culprit drugs that could cause drug reactions such as SJS/TEN. For example, sulfamethoxazole/trimethoprim is frequently prophylactically prescribed to prevent pneumocystis pneumonia, while sulfamethoxazole/trimethoprim is also a common offending drug inducing adverse drug reactions including SJS/TEN. Taylor et al. established a model of GVHD, and described that the activation and proliferation of donor T cells, targeting organs of the recipient, are promoted by proinflammatory cytokines, such as TNF-α and interleukin-1 from recipient macrophages (8). Meanwhile, aGVHD and SJS/TEN shared the similar pathomechanism mediated by cytotoxic T cells (16). Consequently, there are some resemblances in the clinical presentations and histopathology among these two entities. The classic presentations of aGVHD after HSCT, including pancytopenia, diarrhea, and elevated bilirubin, are uncommon in SJS/TEN. Severe pancytopenia and diarrhea in our patient favored the diagnosis of aGVHD, though some medications commonly used after transplantation (mycophenolate mofetil, valganciclovir, and sulfamethoxazole/trimethoprim) and viral infections (HSV, EBV, CMV, and parvovirus B19) may cause cytopenia in rare conditions. Notably, liver function in aGVHD after OLT is usually normal due to the lymphocytes involved in OLT-aGVHD belonging to the liver graft, making a differential diagnosis from SJS/TEN more difficult (3). Moreover, cutaneous manifestations are the most common and often among the earliest findings in both HSCT and solid organ transplantation related aGVHD. Skin rashes in solid organ transplantation-aGVHD can present as erythematous-to-violaceous morbilliform eruptions, which often start on the face, palms, and soles, then extending to the trunk and proximal extremeties (17). SJS-like atypical flat targets (**Figure 1B**) and TEN-like blisters can also present (17). Follicular maculopapules, shown in **Figure 1C**, is a manifestation relatively specific to aGVHD. The main histopathologic findings in aGVHD and SJS/TEN both show vacuolar interface dermatitis and dyskeratotic keratinocytes, and can present as full thickness epidermal necrosis with subepidermal bullae in severe cases. The pathognomic features of aGVHD were satellite necrosis and periadnexal lymphocytic infiltrates, which were not seen in the present case. Thus, it was hard to differentiate aGVHD from SJS/TEN merely based on the histopathology.

Our patient developed skin eruptions 1 month after OLT and 4 days after taking sulfamethoxazole/trimethoprim. GVHD often developes 3 to 5 weeks after OLT, while the induction interval of drug causing SJS/TEN is usually 4 days to 28 days after drug administration (5, 18). Our previous study demonstrated that HLA-B*13:01, HLA-B*15:02, HLA-B*38:02 and HLA-B*39:01 are strongly associated with sulfamethoxazole/trimethoprim-induced severe cutaneous adverse reactions in Asians, including SJS, TEN, and drug reactions with eosinophilia and systemic symptoms (DRESS) (14). In addition, granulysin-based lymphocyte activation tests represent *in vitro* cytotoxic T-lymphocyte memory response to culprit drugs and are useful to confirm drug causality and drug-induced severe cutaneous adverse reactions (13). A low score calculated by ALDEN score of drug causality in SJS/TEN and the Naranjo algorithm (13, 18), a negative lymphocyte activation test for sulfamethoxazole/trimethoprim, and an absence of *HLA-B* risk genotypes helped to exclude the possibility of sulfamethoxazole/trimethoprim-induced SJS/TEN. Last, the donor lymphocyte macrochimerism (19% of HLA originated from the donor) in recipient peripheral blood further confirmed the diagnosis of aGVHD. To solve this diagnostic dilemma, complete physical exmanaition of skin rashes, comprehensive review of drug history, drug causality algorithm, lymphocyte activation test, viral serology test, skin biopsy, and HLA typing of peripheral blood lymphocytes to identify chimerism are mandatory.

Considering the high mortality rates of aGVHD, there have been many attempts of treatment modalities, mainly immunosuppressants. The therapeutic modalities nowadays for aGVHD after HSCT include corticosteroid, anti-thymocyte globulin (ATG), mycophenolate mofetil, sirolimus, anti-T- or B-cell antibodies, cytokine inhibitors, and cellular therapies with variable therapeutic success (20). Corticosteroids are often the first-line therapy because of the ability of profound anti-inflammation effect and lymphocyte apoptosis. However, aGVHD after OLT has been less responsive to corticosteroids (5). The therapeutic results of corticosteroid-refractory aGVHD after HSCT were disappointing with poor survival rates (21) and the novel therapies in OLT-aGVHD remain unexplored. There are no US Food and Drug Administration-approved treatments for OLT-aGVHD currently. Based on the current understanding of the pathogenesis of aGVHD, several cytokines play a key role in the initiation of aGVHD by influencing cytotoxic T-cell differentiation and mediating direct tissue injuries (22). Targeting cytokine inhibitors have emerged as a new option to treat corticosteroid-refractory aGVHD, including TNF-α blockade. TNF-α, secreted by monocytes and macrophages, is associated with the development of aGVHD (17). Serum TNF-α levels are shown to be elevated in HSCT-aGVHD and the therapeutic success of TNF-α inhibitors has been reported in clinical trials and case series (9, 10). However, there has been limited experience of using TNF-α inhibitors in OLT-aGVHD. There have been two TNF-α inhibitors used in HSCT-aGVHD reported, including etanercept, a dimeric fusion protein produced by recombinant DNA, and infliximab, a chimeric monoclonal antibody directed against soluble and transmembrane TNF-α (10). The best responses to infliximab in treating HSCT-aGVHD were noticed in patients without liver involvements, giving a hint of using TNF-α inhibitors in aGVHD following OLT (23). Infliximab was used in a case of OLT-aGVHD refractory to corticosteroids and ATG with successfulness (23). Infliximab binds only to TNF-α, while etanercept targets both TNF-α and TNF-β, which has the same proinflammatory effect as TNF-α (22). Meanwhile, infliximab induces elimination of monocytes and macrophages expressing membrane-bound TNF-α, which may have a higher susceptibility to infections in infliximab users than in etanercept users (24). Furthermore, etanercept was reported to decrease transplanted cell clearance, accelerate liver repopulation, and improve cell engraftment (25, 26).

Rather than the traditional concept of enhancing immunosuppression to treat aGVHD, therapeutic success is seen when withdrawing immunosuppressants (27). It is based on the assumption that decreasing use of immunosuppressants may allow the host immunity to attack reactive donor lymphocytes more effectively. Furthermore, high-dose corticosteroids with profound immunosuppression may aggravate infection (5). In this situation, withdrawing immunosuppressants could not only restore immunological competence, but also prevent morality from overwhelming sepsis. Accordingly, tacrolimus and methylprednisolone were not prescribed at the initial diagnosis of aGVHD and restarted at a low dosage in our case. Anti-TNF-α blockade with etanercept as an initial treatment for aGVHD replaced traditional immunosuppressants to reduce the duration of immunosuppression and thereby lowered the risk of infection. Concerning *E. coli* sepsis and severe pancytopenia, only two doses of 50-mg etanercept were prescribed, and the generalized skin rashes had dramatic improvements in 1 week. Under adequate critical care support, broad-spectrum antibiotics treatment, and protective isolation, sepsis was successfully treated. Then, we started to administrate immunosuppressants and pancytopenia was reversed after giving high-dose methylprednisolone and tacrolimus.

In summary, though there have been no standardized protocols to diagnose aGVHD after OLT, a comprehensive diagnostic evaluation is important to reach the diagnosis. During a critical infection and neutropenia status, adjustment of the traditional immunosuppressants is needed; targeting corticosteroid-sparing biological agent with TNF-α blockade may be an effective alternative treatment for aGVHD after OLT.

## Data availability statement

The original contributions presented in the study are included in the article. Further inquiries can be directed to the corresponding authors.

## Ethics statement

The studies involving human participants were reviewed and approved by Chang Gung Medical Foundation Institutional Review Board (No. 202000622B0). Written informed consent was obtained from the individual for the publication of any potentially identifiable images or data included in this article.

## Author contributions

Y-TH, Y-RC, and H-NW extracted the data from the hospital. Y-TH, Y-RC, C-BC, and C-FL contributed to writing of the case report. C-BC, W-CL, and C-FL supervised the case. All authors contributed to the article and approved the submitted version.

## Acknowledgments

The authors gratefully acknowledge all team members who dedicate their best efforts in taking care of transplant patients.

## Conflict of interest

The authors declare that the research was conducted in the absence of any commercial or financial relationships that could be construed as a potential conflict of interest.

## Publisher’s note

All claims expressed in this article are solely those of the authors and do not necessarily represent those of their affiliated organizations, or those of the publisher, the editors and the reviewers. Any product that may be evaluated in this article, or claim that may be made by its manufacturer, is not guaranteed or endorsed by the publisher.

## Abbreviations

aGVHD, acute graft-versus-host disease; ALDEN, algorithm of drug causality for epidermal necrolysis; ATG, anti-thymocyte globulin; CMV, cytomegalovirus; EBV, Epstein–Barr virus; *E. coli, Escherichia coli;* GI, gastrointestinal; HLA, human leukocyte antigen; HSCT, hematopoietic stem cell transplant; HSV, herpes simplex virus; NSAID, nonsteroidal anti-inflammatory drug; OLT, orthotopic liver transplantation; SJS, Stevens–Johnson syndrome; TEN, toxic epidermal necrolysis; TNF-α, tumor necrosis factor alpha.

## References

[1] Wu GS Selvaggi G Nishida S Moon J Island E Ruiz P. Graft-Versus-Host disease after intestinal and multivisceral transplantation. Transplant (2011) 91(2):219–24. doi: 10.1097/TP.0b013e3181ff86ec 21076376

[2] Burdick JF Vogelsang GB Smith WJ Farmer ER Bias WB Kaufmann SH. Severe graft-Versus-Host disease in a liver-transplant recipient. New Engl J Med (1988) 318(11):689–91. doi: 10.1056/NEJM198803173181107 3278235

[3] Chen WT Kuo TT Kuo KL Yang CS. Graft-Versus-Host disease after orthotopic liver transplantation: A case report and review of the literature. Dermatol Sin (2019) 37(4):213.

[4] Perri R Assi M Talwalkar J Heimbach J Hogan W Moore SB. Graft vs. host disease after liver transplantation: A new approach is needed. Liver Transpl (2007) 13(8):1092–9. 10.1002/lt.21203 17663410

[5] Murali AR Chandra S Stewart Z Blazar BR Farooq U Ince MN. Graft versus host disease after liver transplantation in adults: A case series, review of literature, and an approach to management. Transplant (2016) 100(12):2661–70. doi: 10.1097/TP.0000000000001406 PMC5118135 27495762

[6] Neumann UP Kaisers U Langrehr JM Muller AR Blumhardt G Bechstein WO. Fatal graft-Versus-Host-Disease - a grave complication after orthotopic liver-transplantation. Transplant Proc (1994) 26(6):3616–7. 7998294

[7] Jeanmonod P Hubbuch M Grunhage F Meiser A Rass K Schilling MK. Graft-Versus-Host disease or toxic epidermal necrolysis: Diagnostic dilemma after liver transplantation. Transpl Infect Dis (2012) 14(4):422–6. doi: 10.1111/j.1399-3062.2012.00746.x 22650490

[8] Taylor AL Gibbs P Sudhindran S Key T Goodman RS Morgan CH. Monitoring systemic donor lymphocyte macrochimerism to aid the diagnosis of graft-Versus-Host disease after liver transplantation. Transplant (2004) 77(3):441–6. doi: 10.1097/01.TP.0000103721.29729.FE 14966423

[9] Schmaltz C Alpdogan O Muriglan SJ Kappel BJ Rotolo JA Ricchetti ET. Donor T cell-derived TNF is required for graft-Versus-Host disease and graft-Versus-Tumor activity after bone marrow transplantation. Blood (2003) 101(6):2440–5. doi: 10.1182/blood-2002-07-2109 12424195

[10] Busca A Locatelli F Marmont F Ceretto C Falda M. Recombinant human soluble tumor necrosis factor receptor fusion protein as treatment for steroid refractory graft-Versus-Host disease following allogeneic hematopoietic stem cell transplantation. Am J Hematol (2007) 82(1):45–52. doi: 10.1002/ajh.20752 16937391

[11] Zhang J Lu CW Chen CB Wang CW Chen WT Cheng B. Evaluation of combination therapy with etanercept and systemic corticosteroids for stevens-Johnson syndrome and toxic epidermal necrolysis: A multicenter observational study. J Allergy Clin Immunol Pract (2022). 10(5):1295–304.e6. doi: 10.1016/j.jaip.2022.01.038 35131514

[12] Wang CW Yang LY Chen CB Ho HC Hung SI Yang CH. Randomized, controlled trial of TNF-α antagonist in CTL-mediated severe cutaneous adverse reactions. J Clin Invest (2018) 128(3):985–96. doi: 10.1172/JCI93349 PMC5824923 29400697

[13] Chu MT Wang CW Chang WC Chen CB Chung WH Hung SI. Granulysin-based lymphocyte activation test for evaluating drug causality in antiepileptics-induced severe cutaneous adverse reactions. J Invest Dermatol (2021) 141(6):1461–72.e10. doi: 10.1016/j.jid.2020.11.027 33340500

[14] Wang CW Tassaneeyakul W Chen CB Chen WT Teng YC Huang CY. Whole genome sequencing identifies genetic variants associated with Co-trimoxazole hypersensitivity in asians. J Allergy Clin Immunol (2021) 147(4):1402–12. doi: 10.1016/j.jaci.2020.08.003 32791162

[15] Joo DJ Kim SJ Ju MK Park JP Huh KH Choi JS. Stevens-Johnson syndrome in a liver transplant recipient. Transpl Int (2009) 22(6):667–9. doi: 10.1111/j.1432-2277.2008.00819.x 19144093

[16] Chen CB Kuo KL Wang CW Lu CW Chung-Yee Hui R Lu KL. Detecting lesional granulysin levels for rapid diagnosis of cytotoxic T lymphocyte-mediated bullous skin disorders. J Allergy Clin Immunol Pract (2021) 9(3):1327–37.e3. doi: 10.1016/j.jaip.2020.09.048 33039642

[17] Kim GY Schmelkin LA Davis MDP El-Azhary RA Farrell AM Meves A. Dermatologic manifestations of solid organ transplantation-associated graft-Versus-Host disease: A systematic review. J Am Acad Dermatol (2018) 78(6):1097–101.e1. doi: 10.1016/j.jaad.2017.12.050 29288097 PMC6167008

[18] Sassolas B Haddad C Mockenhaupt M Dunant A Liss Y Bork K. ALDEN, an algorithm for assessment of drug causality in stevens-Johnson syndrome and toxic epidermal necrolysis: Comparison with case-control analysis. Clin Pharmacol Ther (2010) 88(1):60–8. doi: 10.1038/clpt.2009.252 20375998

[19] Hsu TJ Liu KL. Stevens-Johnson syndrome and toxic epidermal necrolysis related to immune checkpoint inhibitors: Two cases and literature review. Dermatol Sin (2022) 38(4):236–9. doi: 10.4103/ds.ds_24_20

[20] Naranjo CA Busto U Sellers EM Sandor P Ruiz I Roberts EA. A method for estimating the probability of adverse drug reactions. Clin Pharmacol Ther (1981) 30(2):239–45. doi: 10.1038/clpt.1981.154 7249508

[21] Rogulj IM Deeg J Lee SJ. Acute graft versus host disease after orthotopic liver transplantation. J Hematol Oncol (2012) 5:50. doi: 10.1186/1756-8722-5-50 22889203 PMC3445845

[22] Rashidi A DeFor TE Holtan SG Blazar BR Weisdorf DJ MacMillan ML. Outcomes and predictors of response in steroid-refractory acute graft-Versus-Host disease. Biol Blood Marrow Transplant (2019) 25(11):2297–302. doi: 10.1016/j.bbmt.2019.07.017 PMC6861661 31325587

[23] Tan Y Xiao H Wu D Luo Y Lan J Liu Q. Combining therapeutic antibodies using basiliximab and etanercept for severe steroid-refractory acute graft-Versus-Host disease: A multi-center prospective study. Oncoimmunology (2017) 6(3):e1277307. doi: 10.1080/2162402X.2016.1277307 28405499 PMC5384382

[24] Piton G Larosa F Minello A Becker MC Mantion G Aubin F. Infliximab treatment for steroid-refractory acute graft-Versus-Host disease after orthotopic liver transplantation: A case report. Liver Transpl (2009) 15(7):682–5. doi: 10.1002/lt.21793 19562700

[25] Levine JE. Implications of TNF-α in the pathogenesis and management of GVHD. Int J Hematol (2011) 93(5):571–7. doi: 10.1007/s12185-011-0803-1 PMC3488873 21384095

[26] Chinnakotla S Smith DM Domiati-Saad R Agura ED Watkins DL Netto G. Acute graft-Versus-Host disease after liver transplantation: Role of withdrawal of immunosuppression in therapeutic management. Liver Transplant (2007) 13(1):157–61. doi: 10.1002/lt.20982 17192857

[27] Viswanathan P Kapoor S Kumaran V Joseph B Gupta S. Etanercept blocks inflammatory responses orchestrated by TNF-a to promote transplanted cell engraftment and proliferation in rat liver. Hepatol (2014) 60(4):1378–88. doi: 10.1002/hep.27232 PMC4176524 24844924

